# Biomechanical and tomographic findings in Brown-McLean syndrome

**DOI:** 10.1016/j.ajoc.2024.102136

**Published:** 2024-07-31

**Authors:** Jaime Guedes, Rodrigo Vilares-Morgado, Rodrigo Brazuna, Alexandre Costa Neto, Denisse J. Mora-Paez, Marcella Q. Salomão, Fernando Faria-Correia, Renato Ambrósio

**Affiliations:** a Rio de Janeiro Corneal Tomography and Biomechanics Study Group, Rio de Janeiro, Brazil; b Department of Ophthalmology, Federal University the State of Rio de Janeiro (UNIRIO), Brazil; c Department of Ophthalmology, Centro Hospitalar Universitário de S. João, Porto, Portugal; d School of Medicine at the University of Minho, Braga, Portugal; e Instituto de Olhos Renato Ambrósio and VisareRIO, Rio de Janeiro, Brazil

**Keywords:** Brown-McLean syndrome, Corneal edema, Corneal tomography, Corneal biomechanics

## Abstract

**Purpose:**

Brown-McLean syndrome (BMS) is a clinical condition characterized by peripheral corneal edema with central corneal transparency. This study aims to document the tomographic and biomechanical characteristics of 3 patients with typical BMS features using the Pentacam® AXL and CORVIS ST® (Oculus Optikgeräte GmbH, Wetzlar, Germany).

**Observations:**

Three cases of BMS are presented. Case 1 involves a 26-year-old male, Case 2 a 55-year-old male, and Case 3 a 74-year-old male. The patients in Cases 1 and 3 had bilateral BMS, while the patient in Case 2 had BMS in the right eye and aphakic bullous keratopathy in the left eye. All three patients were aphakic following cataract surgery. Notably, Cases 1 and 2 were first-degree relatives (son and father), both with bilateral microspherophakia and resultant bilateral aphakia from pediatric cataract surgery. Tomographic analysis revealed a consistent increase in corneal thickness from the center to the periphery in BMS eyes, marked by an abrupt rise in the corneal thickness spatial profile (CTSP) and percentage thickness increase (PTI) curves from the thinnest point towards the periphery. There was no loss of parallel isopachs, no displacement of the thinnest point of the cornea, and no evidence of focal posterior corneal surface depression, typical signs of generalized corneal edema. Biomechanically, BMS eyes exhibited relatively normal corneal stiffness, integrated radius, Ambrósio's relational thickness to the horizontal profile (ARTh), and maximum deformation amplitude ratio at 2mm from the corneal apex (DA ratio). However, the left eye of the patient in Case 2, which had aphakic bullous keratopathy, showed altered biomechanical parameters indicative of a softer cornea with loss of rigidity.

**Conclusions and importance:**

This case series is the first to evaluate the biomechanical and tomographic features of eyes with BMS. Despite the abrupt rise in CTSP and PTI curves from the thinnest point towards the periphery, the relatively normal central corneal biomechanical indices in these BMS eyes are expected when edema is limited to the periphery. These indices become abnormal when there is progression to central corneal edema with bullous keratopathy.

## 1 Introduction

Brown-McLean syndrome (BMS) is a clinical condition characterized by peripheral corneal edema with central corneal transparency. It typically occurs in patients with prolonged aphakia following cataract surgery, particularly intracapsular cataract extraction, but can also happen after procedures such as extracapsular cataract extraction, phacoemulsification, or pars plana lensectomy and vitrectomy. Initially, it was referred to as “Peripheral Corneal Edema After Cataract Extraction”.^1^ In Brown-McLean syndrome, corneal edema typically emerges several years after surgery. It usually impacts the cornea's peripheral 2–3 mm, often beginning at the inferior periphery and, in severe cases, spreading circumferentially. The condition does not affect the conjunctiva, and neovascularization is not observed in the affected cornea.^2^^,^^3^ While most BMS patients have a history of previous intraocular surgery, it can, albeit rarely, affect eyes that have not undergone prior surgery.^4^ These individuals often have coexisting factors like spontaneous lens resorption, lens subluxation, or intermittent angle closure.^4^^,^^5^ There's even a reported case of BMS in a patient with myotonic dystrophy who had not undergone any prior intraocular surgery.^6^ This condition was first described before the era of confocal microscopy, and it was initially believed to be associated with a diseased corneal endothelium. However, more recently, confocal microscopy revealed that patients with BMS have normal, healthy endothelium regarding morphology and cell counts. Therefore, the disease's pathophysiology cannot be solely attributed to the endothelial layer.^7^ Earlier reports suggested that iridodonesis in aphakic patients could lead to intermittent peripheral endothelial abrasion and a superior iridectomy was considered protective for the superior cornea.^3^^,^^5^ However, Almousa et al. and Lim et al. separately reported two patients who developed peripheral corneal edema, mainly in the superior region, despite having undergone intracapsular cataract extraction with a superior iridectomy. Confocal microscopy from the affected area showed normal endothelial counts and cell morphology.^8^^,^^9^ These findings challenge the role of the endothelium in the pathophysiology of this syndrome, suggesting a spectrum of endothelial alterations among patients with BMS clinical features.

The majority of BMS patients are asymptomatic, but a few may experience discomfort, including a foreign body sensation or pain due to ruptured peripheral bullae.^4^ Specular microscopy of the central cornea typically reveals normal endothelial cell counts and morphology since the central cornea is often unaffected. Corneal endothelial decompensation is a relevant differential diagnosis for corneal edema after any intraocular operation. However, in such cases, the condition is due to progressive loss of endothelial pump function, leading to progressive corneal edema, often involving the entire cornea in advanced cases.^10^ In such cases, specular microscopy will likely show reduced cell density of hexagonal endothelial cells, pleomorphism, and polymegathism.^11^ The corneal edema in BMS is often non-progressive, as opposed to cases of corneal endothelial decompensation, and further intraocular surgeries can be performed safely as long as any potential corneal endothelial trauma is minimized, with no subsequent central corneal edema.^10^ The visual axis usually remains clear, and the patient enjoys relatively good and stable corrected distance visual acuity (CDVA). Nonetheless, the peripheral corneal edema induces relevant refractive errors and can be a source of irregular astigmatism.^12^ No definite treatment has been described in the literature, although lubricants such as artificial eye drops may help relieve symptoms of irregular ocular surface. Patients should be educated on symptoms of ocular surface complications, such as corneal abrasion, to seek adequate ophthalmic care promptly.^10^

## 2 Findings

### 2.1 Case 1

**Fig. 1:**
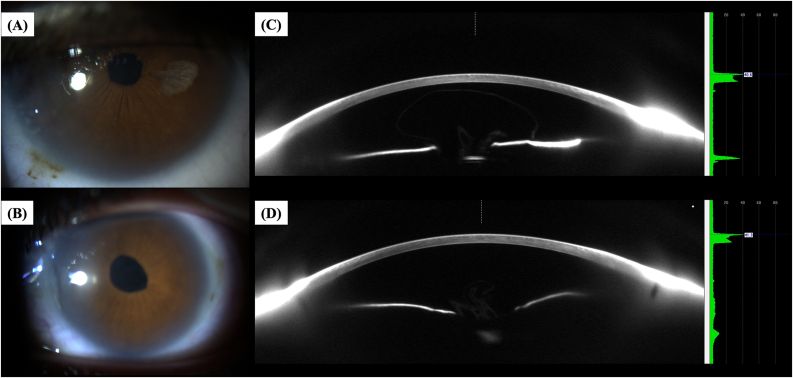
**–** Anterior segment slit lamp photography of the right eye (A) and the left eye (B) of the patient depicted in Case 1. (C) and (D) represent the Scheimpflug photography and densitometry of Scheimpflug images obtained with the Pentacam® AXL (Pentacam®, Oculus Optikgeräte GmbH, Wetzlar, Germany), from the right eye and the left eye of the patient depicted in Case 1, respectively.

**Fig. 2:**
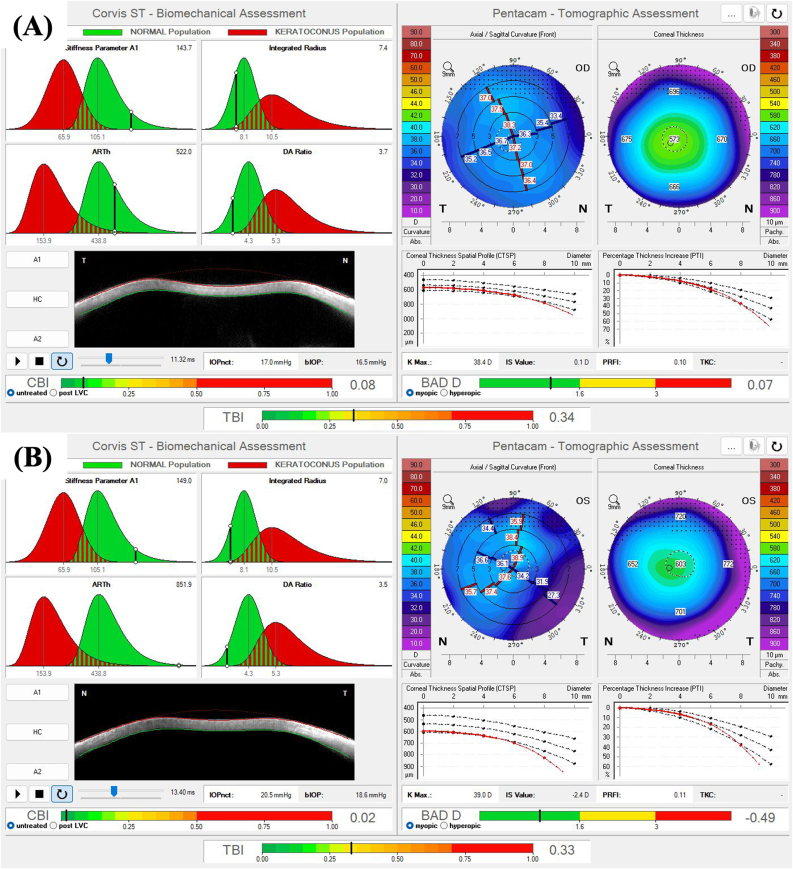
**–** Pentacam® and Corvis® Biomechanical/Tomographic Assessment (Ambrósio, Roberts & Vinciguerra [ARV]) from the right eye (A) and the left eye (B) of the patient depicted in Case 1.

**Fig. 3:**
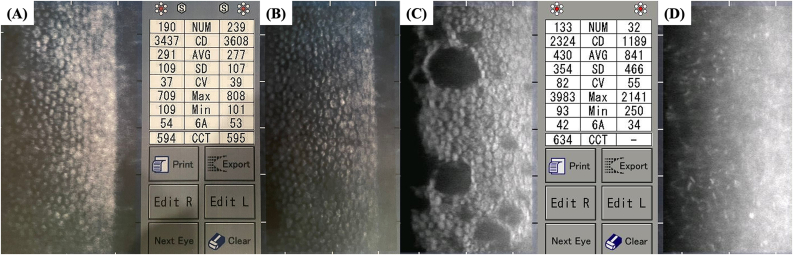
**–** Specular microscopy from the right eye (A) and left eye (B) of the patient depicted in Case 1, as well as from the right (C) and left eye (D) of the patient depicted in Case 2, obtained with the TOMEY EM-3000® (TOMEY GmbH, Nuremberg, Germany).

A 26-year-old male patient presented with isolated peripheral corneal edema in both eyes (OU), persisting for at least six months, leading to peripheral visual field blurring. The patient has a history of microspherophakia, pediatric cataract surgery at age 5, and subsequent aphakia in OU. He also has a central macular scar in the left eye (OS), resulting in functional monocular vision of the right eye (OD). There is a family history of Fuchs’ endothelial dystrophy and microspherophakia. He uses soft monthly contact lenses. Examination showed peripheral microcystic corneal edema with central sparing, central corneal guttata, a tilted optic disc (OD), and a central macular hyperpigmented scar (OS). Scheimpflug imaging revealed characteristic corneal densitometry patterns. Biomechanical and tomographic assessments indicated mild deviations from normal parameters (CBI, BAD-D, TBI). The patient was managed conservatively with prescription renewal, ocular lubrication, and regular follow-up (Table 1, Fig. 1, Fig. 2, Fig. 3).

**Table 1:** Clinical, tomographic, and biomechanical characteristics of the study patients and eyes.

Parameter	Case 1	Case 2	Case 3
Age	26	55	74
Gender	Male	Male	Male
Medical History	Microspherophakia, Pediatric Cataract Surgery (age 5), Aphakia, Central macular scar (OS)	Microspherophakia, Pediatric Cataract Surgery (OD: age 5, OS: age 12), Aphakia, Retinal Detachment (OD)	High Myopia, Cataract Surgery, Retinal Detachment Surgery, Peripheral Field Defects
Family History	Fuchs' Endothelial Dystrophy, Microspherophakia (Father)	Fuchs' Endothelial Dystrophy, Microspherophakia (Son)	Not mentioned
Visual Acuity (UDVA/CDVA)	OD: 20/250, 20/20; OS: HM, HM	OD: Counting fingers, 20/80; OS: 20/200, 20/40	OD: 20/400, 20/40; OS: 20/400, 20/150
Intraocular Pressure (OD/OS)	17 mmHg/18.5 mmHg	13 mmHg/11 mmHg	13 mmHg/12 mmHg
Slit Lamp Findings	Peripheral microcystic corneal edema with central sparing (OU), Central corneal guttata (OU)	Peripheral microcystic corneal edema with central sparing (OD), Central and peripheral bullous keratopathy (OS)	Peripheral microcystic corneal edema with central sparing (OU), Peripheral calcific band keratopathy (OS)
Fundus Examination	Tilted optic disc (OD), Grade 1 fundus tessellation (OU), Central macular hyperpigmented scar (OS)	Tilted optic disc, Grade 2 chorioretinal atrophy, Fundus tessellation (OU), Laser scars (OD)	Tilted optic disc, Grade 3 macular patchy chorioretinal atrophy (OU), Fundus tessellation (OU), Laser scars (OS)
Densitometry (Scheimpflug Images)	One spiking hump with central flattening and a smoothing second hump (OU)	One spiking hump with central flattening and a smoothing second hump (OU)	Increased peripheral corneal thickness (OU), Hyperreflective calcium deposits (OS)
Pentacam & Corvis Biomechanical/Tomographic Assessment	CBI: 0.08, BAD-D: 0.07, TBI: 0.34 (OD); CBI: 0.02, BAD-D: −0.49, TBI: 0.33 (OS)	CBI: 0.05, BAD-D: 0.75, TBI: 0.75 (OD); CBI: 0.76, BAD-D: 0.04, TBI: 0.80 (OS)	CBI: 0.04, BAD-D: 2.90, TBI: 0.86 (OD); CBI: 0.31, BAD-D: 6.38, TBI: 0.95 (OS)
Corneal Thickness (CCT/MCT)	OD: 576 μm/573 μm; OS: 603 μm/599 μm	OD: 618 μm/614 μm; OS: 619 μm/616 μm	OD: 632 μm/631 μm; OS: 634 μm/611 μm
Thinnest point location (relative to corneal apex)	OD: 0.17 mm nasal and 0.25 mm inferior/OS: 0.09 mm nasal and 0.43 mm inferior	OD: 0.60 mm inferior/OS: 0.62 mm nasal and 0.37 mm superior	OD: 0.19 mm nasal and 0.12 mm superior/OS: 0.16 mm temporal and 1.15 mm inferior
Endothelial Cell Density (OD/OS)	3437 cells/mm^2^/3608 cells/mm^2^	2324 cells/mm^2^/Not accurately measurable	3184 cells/mm^2^/2892 cells/mm^2^
Horizontal corneal diameter	OD: 12.0 mm/OS: 11.9 mm	OD: 12.1 mm/OS: 12.6 mm	OD: 11.5 mm/OS: 11.7 mm
Axial Length (OD/OS)	28.22 mm/25.67 mm	29.72 mm/Not measurable	31.13 mm/30.86 mm
Management	Conservative, renewal of prescription, adequate ocular lubrication, regular follow-up	Optimization of ocular surface, lubrication, hypertonic sodium chloride eye drops, proposed OS DMEK	Conservative, lubrication, hypertonic sodium chloride eye drops, OS phototherapeutic keratectomy (PTK)

N/A: Not Available.

### 2.2 Case 2

**Fig. 4:**
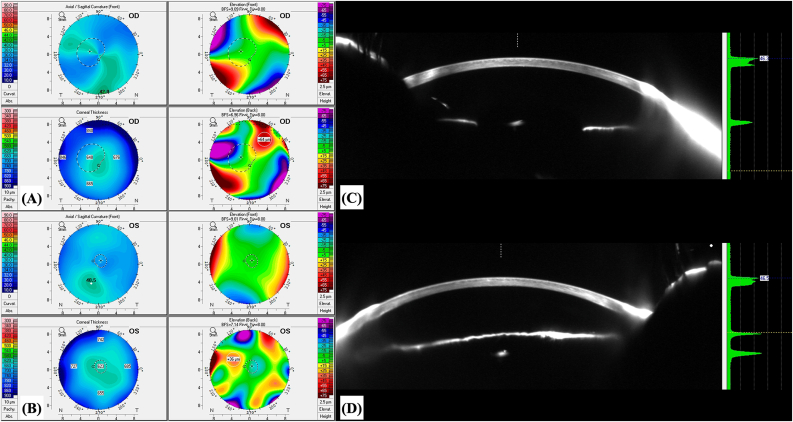
**–** Corneal tomographic parameters of the right eye (A) and the left eye (B) of the patient depicted in Case 2, as well as Scheimpflug photography and densitometry of Scheimpflug images of the right eye (C) and the left eye (D) of the same patient, obtained with the Pentacam® AXL (Pentacam®, Oculus Optikgeräte GmbH, Wetzlar, Germany).

**Fig. 5:**
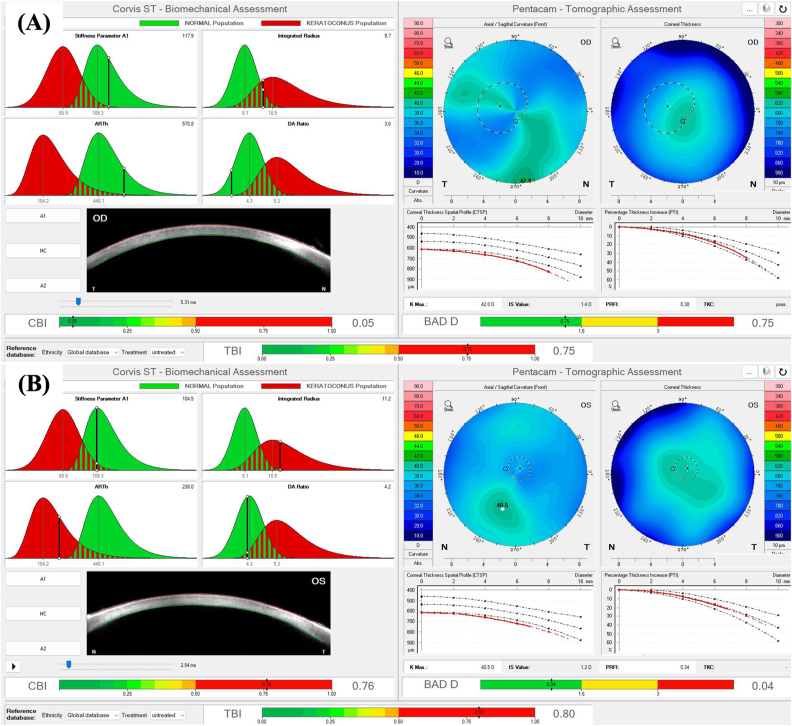
**–** Pentacam® and Corvis® Biomechanical/Tomographic Assessment (Ambrósio, Roberts & Vinciguerra [ARV]) from the right (A) and the left eye (B) of the patient depicted in Case 2.

A 55-year-old male, father of the patient in Case 1, presented with central and peripheral corneal edema in OU, ongoing for two months following a corneal abrasion (OS). He has a history of microspherophakia, pediatric cataract surgery, and subsequent aphakia. He also had a retinal detachment in OD treated with vitrectomy. Examination revealed peripheral microcystic corneal edema (OD), central and peripheral bullous keratopathy (OS), a tilted optic disc, grade 2 chorioretinal atrophy, fundus tessellation, and laser scars (OD). Scheimpflug imaging showed similar densitometry patterns to Case 1. Biomechanical and tomographic assessments revealed moderate abnormalities (CBI, BAD-D, TBI). The patient was managed with ocular surface optimization, lubrication, hypertonic sodium chloride drops, and a proposed DMEK for OS (Table 1, Fig. 4, Fig. 5).

### 2.3 Case 3

**Fig. 6:**
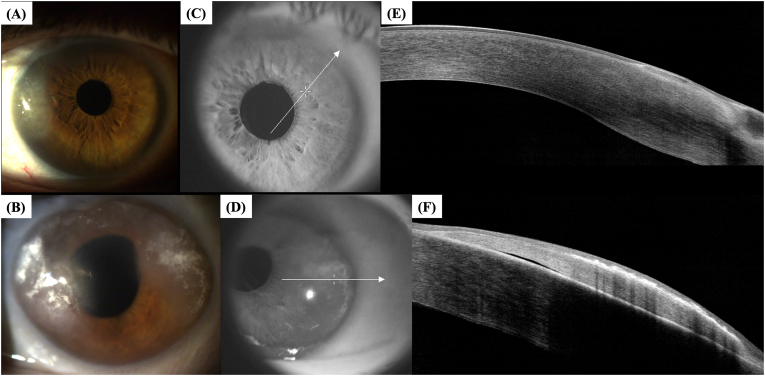
**–** Anterior segment slit lamp photography of the right eye (A) and the left eye (B) of the patient depicted in Case 3. (C) and (D) depict the scan line for the anterior segment optical coherence tomography (AS-OCT), of the right eye and the left eye of the patient depicted in Case 3, respectively. The AS-OCT was performed with the RTVue® (Optovue Inc., Fremont, CA, USA), and is depicted in (E) and (F), for the right eye and the left eye, respectively.

**Fig. 7:**
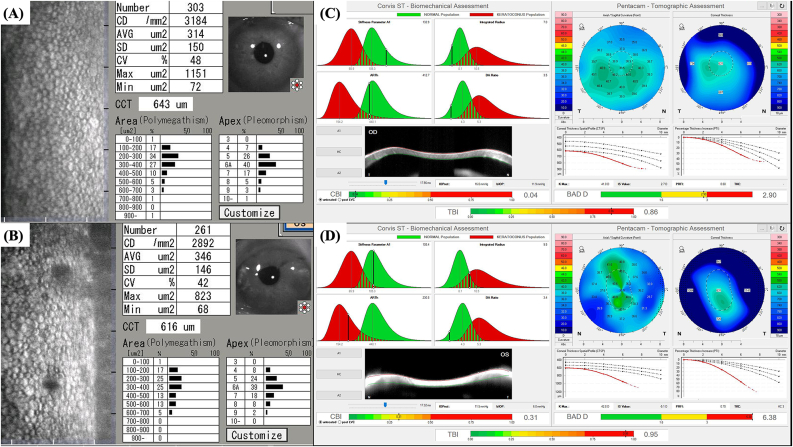
**–** Specular microscopy from the right eye (A) and left eye (B) of the patient depicted in Case 3, obtained with the TOMEY EM-3000® (TOMEY GmbH, Nuremberg, Germany). Pentacam® and Corvis® Biomechanical/Tomographic Assessment (Ambrósio, Roberts & Vinciguerra [ARV]) from the right eye (C) and the left eye (D) of the patient depicted in Case 3.

**Fig. 8:**
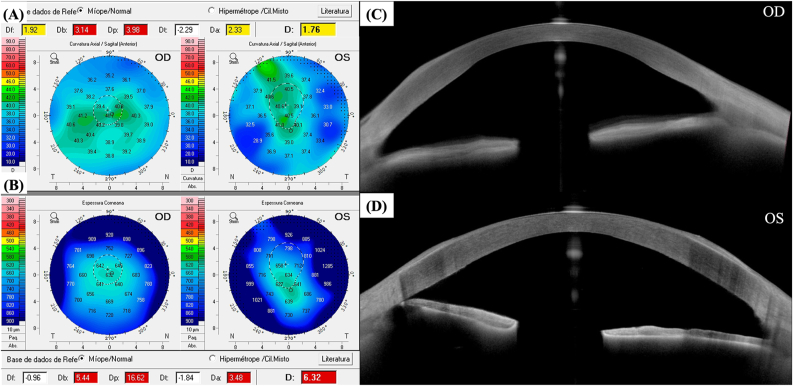
**–** Anterior sagittal curvature maps (A) and corneal thickness maps (B) of the right eye and the left eye of the patient depicted in Case 3, 2 years after undergoing phototherapeutic keratectomy (PTK) in the left eye. (C) and (D) depict the anterior segment optical coherence tomography (AS-OCT), of the right eye and the left eye of the patient depicted in Case 3, respectively. This AS-OCT was obtained with the ANTERION® (Heidelberg Engineering GmbH, Heidelberg, Germany). The improvement in the corneal surface regularity of the left eye following PTK is illustrated in (D).

A 74-year-old male with a complex ophthalmological history of high myopia, cataract surgery, retinal detachment surgery (OS), and peripheral field defects presented with peripheral corneal edema and calcific band keratopathy (OS). Examination showed peripheral microcystic corneal edema with central sparing, a tilted optic disc, grade 3 macular patchy chorioretinal atrophy, fundus tessellation, and laser scars (OS). Anterior segment optical coherence tomography (AS-OCT) indicated increased peripheral corneal thickness and hyperreflective calcium deposits (OS). Biomechanical and tomographic assessments revealed significant abnormalities (CBI, BAD-D, TBI). The patient underwent phototherapeutic keratectomy (PTK) to improve CDVA and corneal aberrations. He showed improvement in CDVA and has been managed with lubrication and regular follow-up for two years (Table 1, Fig. 6, Fig. 7, Fig. 8).

## 3 Discussion

Prior studies have briefly reported tomographic features of BMS using Scheimpflug imaging (Pentacam®, Oculus Optikgeräte GmbH, Wetzlar, Germany). However, to date, no study has reported the tomographic and biomechanical features of eyes with BMS.

In our case series, we present 3 cases of BMS, with two patients presenting bilateral BMS (Case 1 and Case 3). The patient from case 2 presented BMS in OD and aphakic bullous keratopathy in OS. All three patients were aphakic following cataract surgery. Interestingly, patients depicted in Case 1 and Case 2 were first-degree relatives (son and father, respectively), presented bilateral microspherophakia, and underwent pediatric cataract surgery, with resultant bilateral aphakia. The family history of BMS in the first case of our series might imply that BMS can have a genetic etiopathological contribution, and this hypothesis has already been raised in previous BMS studies.^4^ All patients underwent corneal multimodal imaging, with tomographic and biomechanical evaluation.

Regarding tomographic characteristics of the eyes involved in this case series, there was a similar increase in corneal thickness from the center to the periphery, with an abrupt rise in the corneal thickness spatial profile (CTSP) and percentage thickness increase (PTI) curves from the thinnest point towards the periphery. This finding was previously described for keratoconic eyes by Ambrósio et al.^13^ Naturally, unlike keratoconic eyes, eyes presented in this study showed an MCT around at least 570 μm (Table 1), and there were no tomographic signs of corneal ectasia. Furthermore, corneal-thickness spatial profile and percentage increase in thickness curves are also distinct from those observed in eyes with Fuchs’ endothelial corneal dystrophy (FECD) and clinical or subclinical corneal edema, which typically present flatter curves from the thinnest point to towards the periphery.^14^^,^^15^ Eyes with BMS also do not present loss of the parallel isopachs, maintaining their usual disposition, and there is no displacement of the thinnest point of the cornea, which remains close to the corneal vertex, as can be noticed in Table 1 and seen in Fig. 2, Fig. 4, Fig. 6. Finally, as opposed to cases of FECD with subclinical corneal edema, there was no evidence of focal posterior corneal surface depression (initially defined by Sun et al. as any isolated area of depression – negative elevation relative to a sphere with a best-fit zone of 8 mm with float function – within the central 4 mm of the cornea, relative to the pupil center).^15^ The patient depicted in Case 3 presented a bilateral area of focal posterior corneal surface elevation within the central 4 mm of the cornea (relative to a sphere with a best-fit zone of 8 mm with float function), with a maximum elevation of +18 μm in OD and +27 μm in OS. Only one previous study reported tomographic findings in BMS. This was a case report of unilateral BMS by Kam et al.^10^ In their study, the maximum corneal thickness measured on Scheimpflug imaging was 1312 μm, and MCT was 570 μm, located at 1.67 mm nasal and 0.89 mm inferior to the corneal apex, with a maximal corneal density at 4 mm from center in the BMS eye as compared to 6 mm in the fellow eye without BMS. In our study, all three patients (excluding OS from the patient depicted in Case 2, which presented aphakic bullous keratopathy and not BMS) presented CCT and MCT between 570 and 650 μm, with MCT located within the central 2 mm in all eyes (Table 1), as previously reported by Kam et al.^10^

Regarding biomechanical evaluation from our BMS cases performed with the CORVIS ST®, both eyes from patient depicted in case 1 (Fig. 2) presented normal corneal stiffness (defined by the applied pressure divided by the deflection amplitude in the first applanation point- Stiffness Parameter A1),^16^ normal integrated radius (the area under the curve of the corneal inverse radius of curvature (1/central radius of curvature) during the concave phase of deformation over time; softer tissues exhibit smaller radius than stiffer corneas, since more applanation is attained, while a decrease in the integrated radius indicates a stiffening of the cornea),^17^ normal Ambrósio's relational thickness to the horizontal profile (ARTh), which describes the thickness profile in the temporal-nasal direction and is defined as corneal thickness variation from the thinnest point, thus evaluating corneal pachymetric progression,^16^ and a normal maximum deformation amplitude ration at 2mm from the corneal apex (DA ratio), which is calculated based on the ratio between the deformation amplitude (vertical displacement) at the corneal apex and the deformation amplitude at 2 mm nasal and temporal from the apex. Regarding this last biomechanical parameter, the DA ratio is usually higher in softer corneas than in stiffer corneas since, in cases of softer tissues, the cornea starts to deform only in the center, whereas the paracentral part deforms much less. In stiffer corneas, the central and paracentral parts of the cornea are deformed time more homogenously, and the DA ratio is relatively small.^17^ Both eyes from Case 1 presented biomechanical properties that indicate a stiffer cornea (Fig. 2), with high values of Stiffness Parameter A1, low integrated radius, and low DA ratios. Thus, the peripheral corneal edema did not compromise or influence the central corneal biomechanical properties evaluated with the CORVIS ST®. Furthermore, the ARTh was normal in both eyes, implying a normal pachymetric progression from the thinnest point (whose MCT was 573 μm and 603 μm, respectively, for OD and OS) to the corneal periphery. As for the patient depicted in Case 2 (Fig. 5), while OD presented a normal Stiffness Parameter A1, ARTh, and DA ratio, its integrated radius was high, which could be an early sign of variable loss of corneal rigidity and imply an increased risk of progressing to bullous keratopathy, which happened in OS. The OS from the patient depicted in Case 2 presented a borderline Stiffness Parameter A1, an increased integrated radius, a decreased ARTh, and a borderline DA ratio, all signs of a softer cornea with loss of rigidity, which is compatible with the peripheral and central bullous keratopathy with corneal stromal edema, clinically evident in Fig. 4, Fig. 5. OS from Case 2 cannot be considered a typical BMS case since it presents central corneal edema and decreased central corneal endothelial cell density (1189 cells/mm^2^). This eye has probably endured significant trauma to the corneal endothelium during pediatric cataract surgery, and the subsequent corneal abrasion may have further compromised the corneal endothelium, resulting in aphakic bullous keratopathy. Finally, regarding Case 3, while OD presents normal biomechanical indices (Stiffness Parameter A1, integrated radius, and DA ratio), OS presents an increased integrated radius and a more compromised corneal endothelial layer (Fig. 7), which could also be an early sign of variable loss of corneal rigidity and imply an increased risk of progressing to bullous keratopathy. This hypothesis is supported by the significantly decreased ARTh from OS, which means a significant pachymetric progression from the corneal thinnest point to the periphery, which can also be appreciated in CTSP and the PTI curves (Fig. 7).

Nevertheless, even though these tomographic and biomechanical indices may help distinguish decompensated corneas from peripheral corneal edema with sparing of the central endothelium, some of these indices need further study and analysis. Their sensitivity and specificity cannot be fully understood at this point with such a limited number of cases. Furthermore, the second case of this study presents concurrent endothelial dystrophy and BMS, which can confound the accuracy and interpretation of its indices.

Previously, more extensive cohort studies reported a high myopia prevalence of 40 %–61 % in BMS.^3^^,^^12^ In contrast, other large cohort BMS studies found no association with myopia or other refractive errors.^4^ In our study, all three patients presented bilateral high myopia, either confirmed by axial length determination or by fundus evidence of myopic macular degeneration. Furthermore, eyes with BMS typically present an open angle on gonioscopy, while peripheral anterior synechiae can be found occasionally due to surgically induced inflammation.^3^ A previous study by Suwan et al. using ultrasound biomicroscopy (UBM) revealed the absence of iridocorneal touch with widely opened anterior chamber angles.^12^ In our study, all BMS eyes presented an open angle on gonioscopy, which can be confirmed in the AS-OCT of Patient 3 (Fig. 7). Our three patients also demonstrated a long interval between the cataract surgery and the development of clinical evidence of BMS, as previously reported in large BMS cohorts.^4^ The central corneal endothelial cell density was normal in all BMS eyes, with values higher than 2300 cells/mm^2^ (Table 1). This agrees with previous BMS studies, which report normal central corneal endothelial cell density.^2^^,^^9^

## 4 Conclusions

The present case series of 3 cases of BMS, a rare clinical condition characterized by peripheral corneal edema with central corneal transparency, is the first to evaluate biomechanical and tomographic features of eyes with BMS. Even though these eyes present an abrupt rise in CTSP and PTI curves from the thinnest point toward the periphery, their relatively normal central corneal biomechanical indices (Stiffness Parameter A1, integrated radius, and DA ratio) are expected when corneal edema is limited to the periphery (typical of the BMS), becoming abnormal when there is progression to central corneal edema with bullous keratopathy.

## Patient consent

Written consent to publish this case and its details was obtained from all 3 patients.

## Acknowledgments and disclosures

### Funding sources

No funding or sponsorship was received for this study or publication of this article.

## Authorship

All named authors meet the International Committee of Medical Journal Editors (ICMJE) criteria for authorship for this article, take responsibility for the integrity of the work as a whole, and have given their approval for this version to be published.

## Data availability statement

This article is a case series. Data sharing is not applicable to this article as no datasets were generated or analyzed during the current study.

## Compliance with ethics guidelines

The patients reported in this case series signed an informed consent form. This study is in accordance with the canons of the Declaration of Helsinki for research involving human participants.

## CRediT authorship contribution statement

**Jaime Guedes:** Writing – original draft, Visualization, Validation, Supervision, Software, Resources, Project administration, Methodology, Investigation, Funding acquisition, Formal analysis, Data curation, Conceptualization. **Rodrigo Vilares-Morgado:** Writing – original draft, Visualization, Validation, Supervision, Software, Resources, Project administration, Methodology, Investigation, Funding acquisition, Formal analysis, Data curation, Conceptualization. **Rodrigo Brazuna:** Writing – original draft, Visualization, Validation, Supervision, Software, Resources, Project administration, Methodology, Investigation, Funding acquisition, Formal analysis, Data curation, Conceptualization. **Alexandre Costa Neto:** Writing – original draft, Visualization, Validation, Supervision, Software, Resources, Project administration, Methodology, Investigation, Funding acquisition, Formal analysis, Data curation, Conceptualization. **Denisse J. Mora-Paez:** Writing – original draft, Visualization, Validation, Supervision, Software, Resources, Project administration, Methodology, Investigation, Funding acquisition, Formal analysis, Data curation, Conceptualization. **Marcella Q. Salomão:** Writing – original draft, Visualization, Validation, Supervision, Software, Resources, Project administration, Methodology, Investigation, Funding acquisition, Formal analysis, Data curation, Conceptualization. **Fernando Faria-Correia:** Writing – original draft, Visualization, Validation, Supervision, Software, Resources, Project administration, Methodology, Investigation, Funding acquisition, Formal analysis, Data curation, Conceptualization. **Renato Ambrósio:** Writing – original draft, Visualization, Validation, Supervision, Software, Resources, Project administration, Methodology, Investigation, Funding acquisition, Formal analysis, Data curation, Conceptualization.

## Declaration of competing interest

The authors have no conflict of interest.
